# Stimulation of α7 Nicotinic Acetylcholine Receptor by Nicotine Suppresses Decidual M1 Macrophage Polarization Against Inflammation in Lipopolysaccharide-Induced Preeclampsia-Like Mouse Model

**DOI:** 10.3389/fimmu.2021.642071

**Published:** 2021-04-28

**Authors:** Xinjia Han, Wei Li, Ping Li, Zheng Zheng, Baohua Lin, Bei Zhou, Kaimin Guo, Ping He, Jinying Yang

**Affiliations:** Department of Obstetrics, Guangzhou Women and Children’s Medical Center, Guangzhou Medical University, Guangzhou, China

**Keywords:** α7nAChR, nicotine, decidual macrophage, placenta, preeclampsia

## Abstract

Changes in decidual macrophage polarization affect local inflammatory microenvironment and lead to adverse pregnancy outcomes. However, the regulatory mechanism of macrophage polarization in preeclampsia (PE) remains unclear. In this study, we found that α7nAChR expression was significantly down-regulated in decidual macrophages in PE patients compared to normal pregnant women, accompanied by a reduced proportion of M2 phenotype and an increased proportion of M1 phenotype; these results suggested that the reduced α7nAChR activity might contribute to changes in the polarization of decidual macrophages. Then, we further investigated the regulatory role of α7nAChR activation by nicotine on decidual macrophage polarization and placental remodeling in the PE-like mouse model. The PE mice were obtained by i.p. injection of 10 µg/kg lipopolysaccharide (LPS) gestational day (GD) 13, and 40 µg/kg LPS daily until GD16. Subcutaneous injection of 1.0 mg/kg nicotine was administrated from GD14 to GD18. Nicotine treatment increased the decreased M2 phenotype and inhibited the increased M1 phenotype in decidua of pregnant mice induced by LPS. The levels of pro-inflammatory cytokines in decidua were higher but the levels of anti-inflammatory cytokines were lower in PE mice compared to the controls, nicotine reversed these changes. The level of choline acetyltransferase (CHAT) was reduced in the LPS-treated group, it was increased following nicotine treatment. Damage of spiral artery remodeling and down-regulation of markers related to trophoblast invasion in placentas were found in PE mice; nicotine improved these pathological structures of placentas. α-bungarotoxin (α-BGT) which is specific antagonist for α7nAChR could abolish the effects of nicotine on decidual macrophage polarization, trophoblast arrangement and vascular structure in placental tissue in PE mice. These results suggest that α7nAChR plays an important regulatory role in maternal-fetal inflammation and placental remodeling in preeclampsia and may provide a theoretical basis for the discovery of new strategies for preeclampsia.

## Introduction

Preeclampsia (PE) is a specific disorder of pregnancy that is characterized by hypertension, proteinuria and other symptoms including headache, vomiting, kidney and liver dysfunction (1, 2). PE is the major cause of maternal and perinatal death; its pathogenesis has not been revealed clearly. Abnormal maternal-fetal immune response plays an important role in the occurrence and development of preeclampsia (3). In PE patients, the number of circulating leukocytes, neutrophils, and serum levels of tumor necrosis factor-α (TNF-α), interleukin-6 (IL-6), C-reactive protein (CRP) were significantly increased compared to normal pregnancy (4). In lipopolysaccharide (LPS)-induced PE-like animal model, the higher levels of TNF-α, IL-6 and monocyte chemokine-1 (MCP-1) correlated with insufficiency of placental spiral artery remodeling (5, 6). LPS also could increase the secretion of IL-6 and IL-8 from extravillous trophoblast (EVT) but decrease the invasion of EVT (7). Elevated concentrations of TNF-α directly impaired trophoblast invasion in first-trimester villous explant cultures (8). These findings suggest that excessive inflammatory state at maternal-fetal interface inhibits trophoblast invasion and contributes to the development of PE. However, the specific mechanisms mediating this process are still under investigation.

Decidual macrophages (dMφ) are the second abundant immune cells in pregnancy (9), the imbalance of dMφ polarization is involved in complications of pregnancy such as PE (10), miscarriage (11), and fetal growth restriction (12, 13). In PE patients, the levels of IL-10 in decidua and CD163 in CD14^+^ dMφ was remarkably lower compared with normal pregnancies (10). It was found that activated peripheral blood macrophages induced by LPS decreased the invasiveness of HTR8 trophoblast (7). Thus, dMφ dysfunction affected trophoblast invasion. The mechanisms regulating dMφ polarization in PE have not been fully revealed.

Epithelial mesenchymal transition (EMT) plays a key role in tumor invasion and metastasis. During EMT, the E-cadherin (the epithelial marker) expression is decreased, and vimentin (the mesenchymal protein) expression is increased (14). It has been shown that α7 nicotinic acetylcholine receptor (α7nAChR) regulates EMT and affects tumor invasion. Feng et al. found that α7nAChR was highly expressed in human cholangiocarcinoma tissue; knockdown of α7nAChR *in vitro* inhibited the EMT process and reduced the migration and invasion of cholangiocarcinoma cells (15). Trophoblasts have the similar invasiveness ability to tumor cells; their invasion process is regulated precisely in the establishment and maintenance of pregnancy (16). Studies from Brown et al. showed that the expression levels of E-cadherin in trophoblasts of placental tissue were significantly increased in preeclamptic women than that in normal pregnant women, suggesting that the EMT process of trophoblasts was damaged in PE, which led to the decrease of their invasive ability (17). However, whether α7nAChR participates in regulating trophoblast invasion in PE is unknown.

Our previous studies found that nicotine (agonist for α7nAChR) could decrease the inflammatory cytokines levels in serum and placenta in LPS-induced PE-like murine model; and improve the clinical symptoms of PE (18). The results suggested that α7nAChR activation might be protective in PE. As the main receptor of cholinergic anti-inflammatory pathway, α7nAChR is widely expressed on macrophages. We hypothesized that α7nAChR might improve the inflammatory response at the maternal-fetal interface in PE by regulating the polarization state of dMφ.

## Materials and Methods

### Study Population

15 normotensive pregnant women served as controls; and 20 preeclamptic women were included in the study. The diagnostic criteria of preeclampsia were as following: >2.0 g proteinuria in 24 h urine collection, systolic/diastolic blood pressure >140/90 mmHg (19–21). Pregnant women with pre-existing renal diseases, diabetes, chronic hypertension infectious, cardiovascular disease, prior preeclampsia, illicit drug use, multiple gestations were excluded. Demographic data of the study population are shown in **Table 1**. The Ethics Committee of the Guangzhou women and children’s medical center approved the study (No. 2018041701 and No. 201922200). Informed consent was obtained from all women. The time of collecting population was from 2018.08 to 2019.12.

**Table 1:** Demographic and biophysical characteristics of the clinical study groups.

Parameter	Healthy women (n=15)	Preeclampsia women (n=20)
Maternal age (years)	28 (23-34)^a^	30 (22-38)
Gestational age (weeks)	39 (37-41)^a^	37 (32-39)^*^
SBP(mmHg)	113 ± 9.1	158 ± 18.6^*^
DBP(mmHg)	75 ± 2.8	103 ± 7.3^**^
Urine protein (g/24 h)	0	2.9 ± 1.1^***^
The percentage of caesarean	16.7%	89.0%^***^

a The numbers are median and range unless otherwise indicated.

Blood pressure values are presented as mean ± SEM, student’s t test is used to compare differences between groups. Mode of delivery are presented as %, the Mann–Whitney test is used to test for differences between groups. ^*^P < 0.05, ^**^P < 0.01, and ^***^P < 0.001 compared to normal pregnancy.

### Sample Collection

All placental tissues were obtained within 10 min of delivery by cutting a vertical plane spanned from the fetal membranes to the decidua. Then tissues were quickly rinsed in 0.9% saline and decidual samples were dissected out under an anatomical microscope using a 20 cm ruler; 5x5 mm^2^ decidual tissues were fixed in 4% paraformaldehyde (at 4°C for 2-3 days) (20, 22) and embedded with paraffin, and made into 4 µm-thick sections for immunohistochemistry.

### Animals and Mouse Model of PE

All procedures were approved by the Guangzhou Medical University Animal Ethics Committee (Permit Number: 2012–50) and strictly performed in accordance with the National Institute of Health Guide for the Care and Use of Laboratory Animals. 10-12 weeks old of adult C57BL/6 mice were purchased from the Medical Experimental Animal Centre of Guangdong, China. After a week of acclimatization, all female mice were mated with healthy males at a 2:1 ratio, and a positive vaginal smear for sperm defined gestational day (GD) 0. Pregnant mice were randomly divided into four groups (10 mice each): pregnant control group (P), LPS-treated group (LPS), LPS and nicotine-treated group (LPS+N), LPS, nicotine and α-BGT-treated group (LPS+N+A). The experimental PE mice were induced by intraperitoneal injection of LPS (10µg/kg body weight, dissolved in 0.5 mL saline) on GD 13, followed by 40µg/kg daily until GD16 (6, 23). Subcutaneous injection of 1 mg/kg nicotine per day at 9:00 a.m. and 3:00 p.m. from GD 14 to 18 was carried out in two sessions; the administration of nicotine was 30 min before LPS injection. 1µg/kg α-BGT was administrated subcutaneously in two sessions and each session was 30 min prior to nicotine (24).

### Histology and Immunofluorescent Staining

On GD18, the pregnant mice were anesthetized with 10% chloral hydrate (3 ml/kg, intraperitoneal injection), the mouse placental tissues and decidual tissues were fixed in 4% paraformaldehyde (at 4°C for 2-3 days) and embedded with paraffin; and made into 4 µm-thick sections. For hematoxylin and eosin (H&E) staining, 6-8 random areas were captured in each section at the same magnifications (6 sections for each sample, n=5) under a Leica DM4B microscope (Wetzlar, Germany). The number of cytotrophoblasts (CTBs) and syncytiotrophoblasts (STBs) were separately counted in a trophoblastic layer unit (floating villi, FV) containing vascular lumen using Image J (National Institutes of Health, NIH), values from 6 sections of each sample were averaged; and such mean number were used to be compared between two groups. The length of vascular lumen in a trophoblastic layer was measured using Image J with a unit of 0.487μm/pixel, the measurement was carried out in three equal intervals along the long axis of the vascular lumen, and the 3 values were averaged, and the statistical analysis followed that of the number of CTBs and STBs.

Both the human and mouse decidual slices were preheated at 60°C for 1 h. Following deparaffinization and rehydration with ethanol, sections were immersed in 1% H_2_O_2_ to block the nonspecific antigens and endogenous peroxidase activity. After washed in 0.01M PBS, sections were incubated at room temperature for 2 h with 5% normal goat serum diluted in 0.3% triton in PBS. Primary antibodies incubation was processed at 4°C for 20 hrs, the information for single immunofluorescent staining was as following: anti-MMP-9 (rabbit, 1:100, cat#AF5228, Affinity Biosciences, Cincinnati, OH, USA), anti-α-SMA (rabbit, 1:100, cat#bs-10196R, Bioss Biotechnology, Beijing, China); the information for double immunofluorescent staining was as following: α7nAChR (rabbit, 1:50, cat# ab10096, Abcam, Cambridge, MA, USA), CD68 (mouse,1:50, cat# ab955), CD86 (rabbit, 1:50, cat# bs1035R, Bioss Biotechnology), CD163(rabbit, 1:50, cat# ab182422), anti-E-cadherin (mouse, 1:40, cat#sc-8426, Santa Cruz Biotechnology, Dallas, TX, U.S.A.), anti-cytokeratin 7 (CK7, rabbit, 1:20, cat# bs-1744R, Bioss Biotechnology).Sections were then rinsed in PBS and incubated with a secondary antibody for 3 hrs at room temperature. Secondary antibodies were goat anti-rabbit (1:500, cat# ab150081, Alexa Fluor 488), goat anti-mouse (1:500, cat# ab150117, Alexa Fluor 488), goat anti-rabbit (1:500, cat# ab150088, Alexa Fluor 594). After washing with 0.01M PBS, sections were stained with DAPI to identify the nucleus. Images were acquired using the same light intensity under a fluorescence microscope (DM4B, Leica, Wetzlar, Germany).

For analyzing of immunostaining results of MMP-9, α-SMA and E-cadherin, 18 sections from each group (3 sections for each animal, n=6) were used. The fluorescence intensity was measured using Image J (NIH) and figures were processed with threshold adjustment. The results were expressed relative to the data from the control group. For the immunolocalization analysis in human and mouse decidua, all intracellular puncta within each figure were selected and analyzed using the JACoP plugin of ImageJ software (NIH) (25, 26). 15 sections from each group (3 sections for each sample, n=5) were used. The results were expressed relative to the data from the control group.

### Preparation of Mouse Cells and Flow Cytometry (FCM)

On GD18, decidual tissues from anesthetized pregnant mice were dissected out from the fetal and placental tissues, then quickly washed in ice-cold PBS and minced and digested in RPMI 1640 (HyClone, U.S.A.) supplemented with collagenase type IV (1.0mg/ml, cat# 9001-12-1, Gibico, Thermofisher scientific) and DNase I (150U/ml, cat# 1121MG010, Biofroxx, Germany) for 2 h at 37°C with gentle agitation (27). The digestion process was terminated by double volume ice-cold of RPMI 1640, and the cell suspensions were filtered through a 100 μm cell strainer and centrifuged at 1500 rpm for 5 min at 4°C, then red blood cells in the enriched cells were removed by red blood cell lysis. Cell suspension was washed in PBS twice and resuspended in staining buffer for FCM. The isolated cells were stained with Alexa Fluor 647 anti-mouse F4/80, PE anti-mouse TNF-α, FITC anti-mouse CD206, PE anti-mouse CD86, PE anti-mouse CD163, FITC anti-mouse IL-10, PE-Cy7 anti mouse IL-1β at 4°C in the dark for 30 min and washed in PBS twice. FACScanto™ II instrument (BD Biosciences) was used for analysis; gating was set to a minimum of 10,000 viable cells and analyzed by forward and side scattering.

For analysis of the intracellular cytokines iNOS and Arg-1, firstly isolated cells were stained with Alexa Fluor 647 anti-mouse F4/80 at 4°C in the dark for 30 min. After washing with staining buffer twice, cells were fixed and permeabilized using a Fixation/Permeabilization Solution Kit (BD Biosciences, United States) for 1h at 4°C in the dark. After washing twice, cells were incubated with PE anti-mouse Arg-1, PE anti-mouse iNOS at 4°C in the dark for 45 min and then washed twice.

For analysis the expressions of CHAT on macrophages, after being incubated with Alexa Fluor 647 anti-mouse F4/80 mAbs at 4°C in the dark for 30 min, cells was washed twice. Then cells were stained with anti-CHAT (rabbit, 1:100, cat# ab181023) antibody at 4°C in the dark for 30 min and washed twice. The cells were staining with Alexa Fluor 488 goat anti-rabbit (1:200, cat# ab150081) at 4°C in the dark for 35 min and then washed twice. Data are presented as means ± SD.

### Statistical Analysis

Data are presented as mean ± SEM. Differences between groups were analyzed by student’s t-test. Differences among multiple groups were analyzed using one-way ANOVA. Statistical analyses were conducted with SPSS v17.0 (SPSS, Chicago, IL, USA). P *<* 0.05 was used to determine statistical significance.

## Results

### PE Is Associated With a Striking Down-Regulation of α7nAChR on (dMφ) and the Imbalance of dMφ Polarization

**Figure 1:**
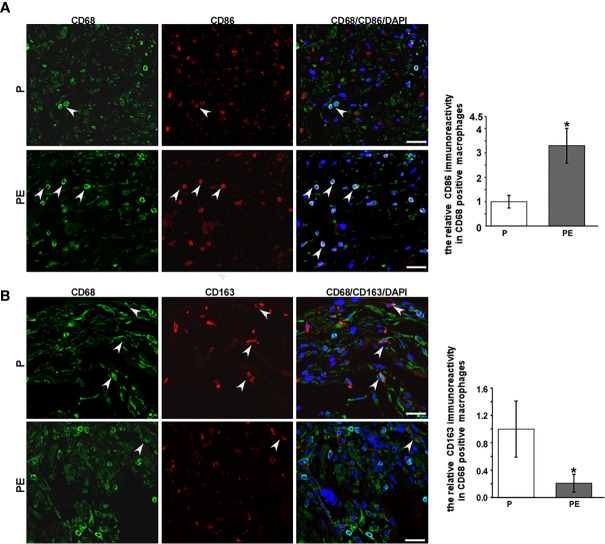
A higher expression of CD86 on decidual macrophages (dMφ) with a lower expression of CD163 on dMφ was found in PE patients. **(A)** Representative immunofluorescence images of CD86 (M1 biomarkers) on dMφ in PE patients and normal controls; the relative CD86 positive CD68 immunoreactivity was quantified as shown in the graph. CD68 was stained as pan-macrophage biomarker. White arrows indicated positive stained signals. **(B)** Representative immunofluorescence images of CD163 (M2 biomarkers) on dMφ in PE patients and normal controls; quantification for the relative CD163 positive dMφ immunoreactivity was shown in the graph. White arrows indicated positive stained signals. Scale bar=30 μm.^*^P *<* 0.05.

**Figure 2:**
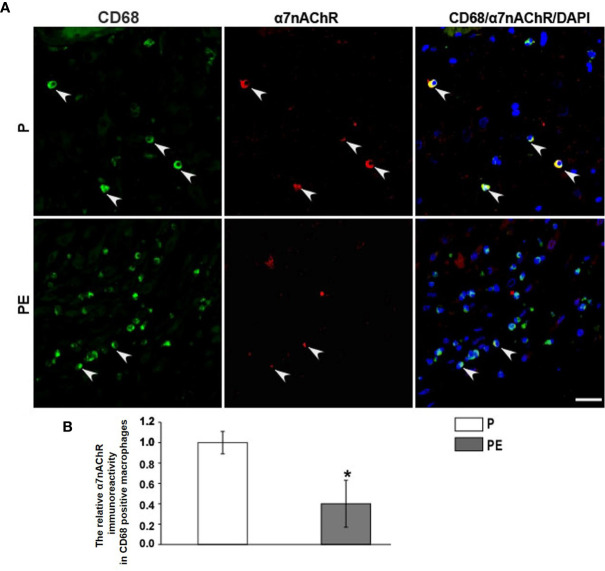
PE is associated with a striking down-regulation of a7nAChR on decidual macrophages (dMj). **(A)** Representative immunofluorescence images of a7nAChR on dMj in PE patients and normal controls. White arrows indicated positive stained signals. **(B)** The relative a7nAChR positive dMj immunoreactivity was quantified as shown in the graph. Scale bar=30 mm. *P < 0.05.

The immunofluorescent staining of CD68 (pan-macrophage marker), CD86 (M1 marker), CD163 (M2 marker) was performed in the decidua of PE patients and normal pregnant women. The dMφ phenotype in patients presented an M1 predominance (**Figure 1A**), but the M2 dMφ decreased significantly (**Figure 1B**); which demonstrated the disturbance of maternal–fetal immunoregulation in PE. In addition, compared with normal pregnancy (P), α7nAChR expression on dMφ in PE patients was remarkably decreased (**Figure 2**). The cholinergic anti-inflammatory pathway is mainly mediated by α7nAChR on macrophage (28). Thus, the imbalance of dMφ polarization in PE might be caused by inhibition of α7nAChR-mediated cholinergic anti-inflammatory pathway, finally induced excessive inflammation and contributed to the pathology of PE.

### α7nAChR Stimulation by Nicotine Greatly Improved Placental Injury in Pregnant Mice Induced by LPS

**Figure 3:**
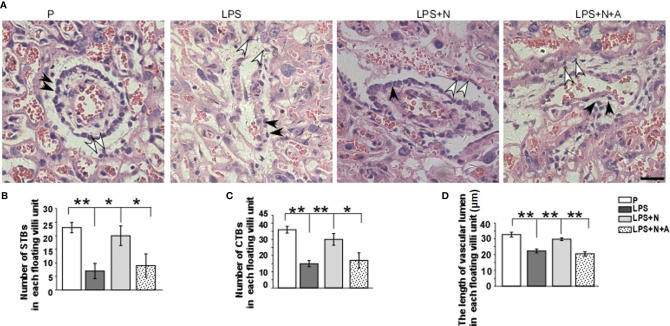
H&E staining showed morphological changes of trophoblasts and vascular lumen in placentas from all experimental groups. **(A)** White arrows indicated syncytiotrophoblasts (STBs), black arrows indicated cytotrophoblasts (CTBs). The number of STBs and CTBs seemed decreased in LPS group, their shape seemed being deformed; vascular lumen also became narrow. The α7nAChR agonist (nicotine) alleviated pathological changes of placenta to some extent. Histograms showed changes in the number of STBs **(B)** and CTBs **(C)** and the length of vascular lumen **(D)** in the placentas in all animal groups. Scale bar=30 μm. ^*^P *<* 0.05 and ^**^P *<* 0.01.

To investigate whether regulating α7nAChR activity can alter placental injury in PE, H&E staining was used to detect the morphological changes in the placenta in all animal groups. In normal pregnant mice, the trophoblasts seemed normal in shape and arranged in order. In the LPS-treated mice, the number of STBs and CTBs became less and loose, their shapes became irregular; the vascular lumen became narrower than that in the P group (**Figure 3A**). Following nicotine administration, the shape, number and arrangement of trophoblast and the shape of vascular lumen were restored to some extent. The α7nAChR antagonist reversed the effects of nicotine. The number of CTBs and STBs in each floating villi unit were counted using Image J (**Figures 3B, C**); the length of vascular lumen was also measured (**Figure 3D**). Quantitative analysis showed a similar changing tendency in all groups to that observed from the HE staining images. These data suggest that activating α7nAChR-mediated cholinergic anti-inflammatory pathway can reduce LPS-induced placental injury in pregnant mice.

### α7nAChR Stimulation by Nicotine Played an Effective Role in Ameliorating the Abnormality of Placenta Implantation and Spiral Artery Remodeling in LPS-Induced PE-Like Model

**Figure 4:**
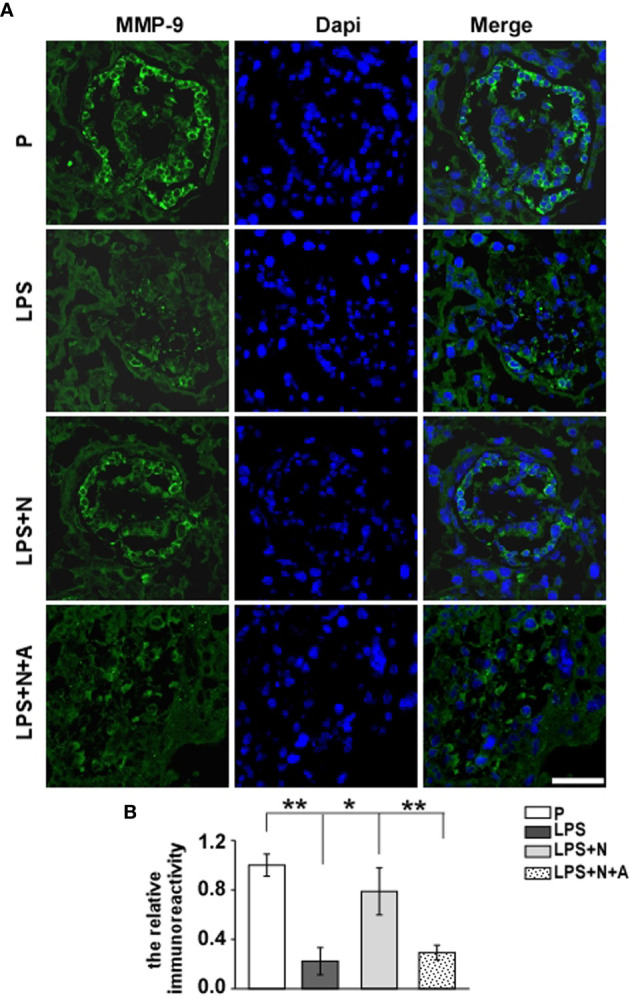
Nicotine up-regulated the decreased MMP-9 expression in placentas from LPS-induced PE-like mice. Representative immunofluorescent images **(A)** and graph bar **(B)** showed changes in levels of MMP-9 immunoreactivity (green) in the trophoblast layer of placentas from all animal groups. Scale bar=50 μm. ^*^P *<* 0.05 and ^**^P *<* 0.01.

**Figure 5:**
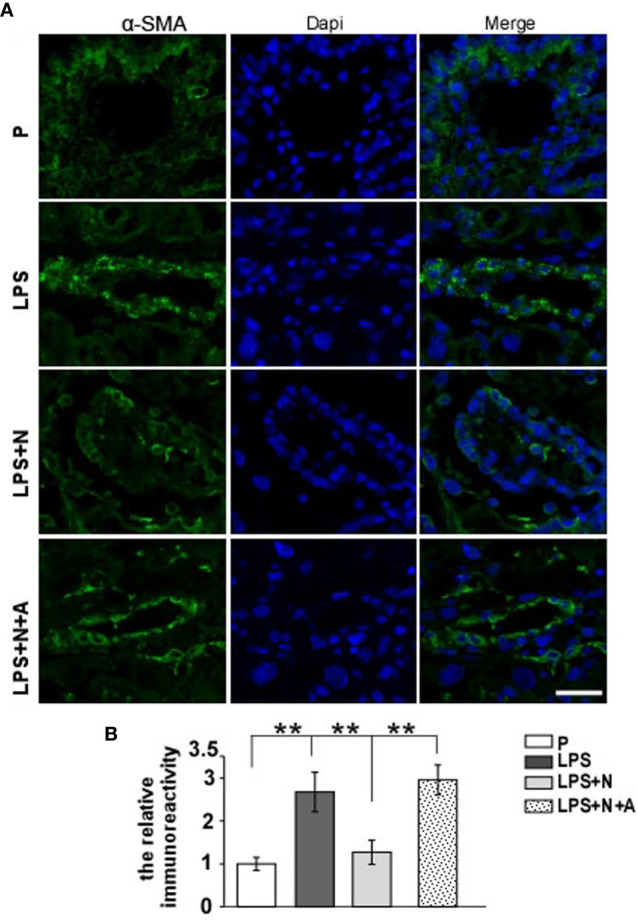
Nicotine reduced the elevated expression of α-SMA and improved the impairment of spiral artery in placentas from the PE-like mice treated by LPS. **(A)** Representative immunofluorescent images for α-SMA in placentas from all experimental groups. Scale bar=30 μm. **(B)** Quantitative analysis showed changes in the relative immunoreactivity of placental α-SMA in all groups. ^**^P *<* 0.01.

**Figure 6:**
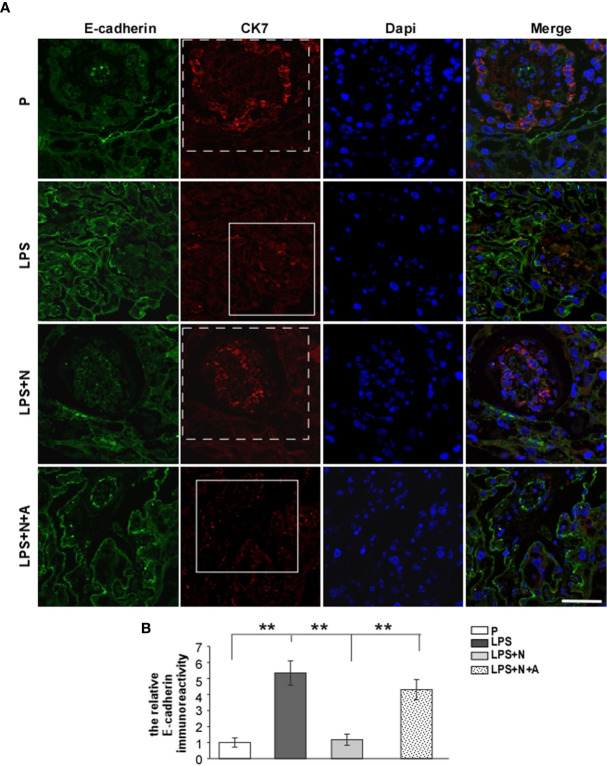
The higher E-cadherin expression in placentas from PE-like mice which indicated invasive abnormity of the trophoblast was inhibited by nicotine. Representative immunofluorescent images **(A)** and quantitative analysis **(B)** showed changes in the relative immunoreactivity of placental E-cadherin in all groups. The dotted frame represented the trophoblast layer stained by CK7 in the P and LPS+N group; the solid frame represented the degenerated trophoblast layer weakly stained by CK7. Scale bar=50 μm. ^**^P *<* 0.01.

It has been reported that the MMP-9 level decreased significantly in the placenta of PE patients compared with normotensive pregnant women (29, 30). And such lower MMP-9 level closely correlated with insufficient invasion of trophoblasts. Thus, we detected changes in the MMP-9 expression in the placenta in all animal groups by immunofluorescent staining to investigate whether nicotine can restore placental dysfunction. In the normal pregnancy group, strong MMP-9 staining was detected on CTBs column according to the cell arrangement shown by dapi; less MMP-9 immunoreactivity in the trophoblasts was observed in the LPS-treated group than in the P group (**Figure 4**). Such decreased immunoreactivity was enhanced following nicotine treatment, which was reversed by α-BGT. At the same time, the alpha-smooth muscle actin (α-SMA) immunoreactivity was increased significantly around the area of disorganized trophoblasts in the LPS-treated group compared to the P group (**Figure 5**), which suggested abnormal spiral artery remodeling. In the nicotine and LPS-treated group (LPS+N), such immunoreactivity was suppressed, and the arrangement of trophoblasts was relatively regular in the region where the α-SMA expression decreased. In addition, we further detected the E-cadherin expression in the placenta and the trophoblasts were labeled by the CK7 antibody. The CK7 staining was strong in CTB columns in the normal pregnancy group, the CK7-positive trophoblasts were unable to react with anti-E-cadherin; E-cadherin was barely expressed on the placental cells which surrounded the trophoblast layer. Higher E-cadherin was present in trophoblasts of PE patients (17). We also found that LPS induced an increase in E-cadherin immunoreactivity which was adjacent to the degenerated trophoblasts in the placenta in pregnant mice (**Figure 6**). Such increased E-cadherin expression was dramatically inhibited by nicotine, the up-regulation of CK7 expression on CTBs column was also found in the LPS and nicotine-treated group. The effects of nicotine could be largely abolished by α-BGT. Therefore, α7nAChR stimulation by nicotine could ameliorate placental dysfunction.

### Nicotine Activated Downregulation of α7nAChR-Mediated Cholinergic Anti-Inflammatory Pathway Induced by LPS in Pregnant Mice

**Figure 7:**
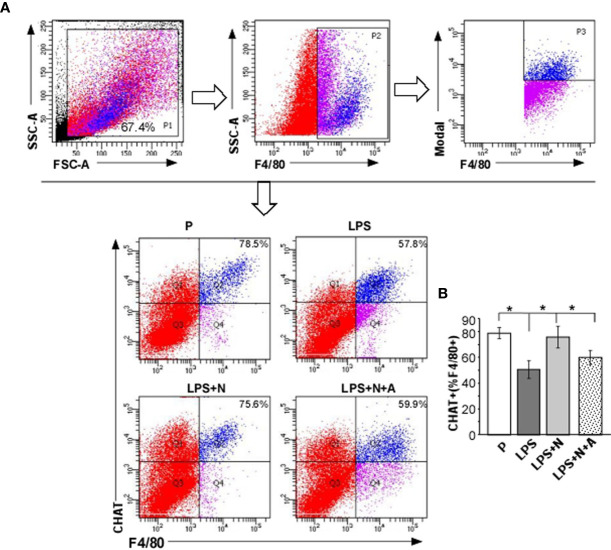
Nicotine upregulated the decreased α7nAChR activity (CHAT expression) on dMφ from LPS-induced PE-like mice. **(A, B)** Percentages of CHAT^+^ dMφ in all experimental groups were calculated by flow cytometric analysis. Total leukocytes were gated using FSC vs. SSC and then gated for identifying CD68^+^ macrophages. Representative data were derived from separate mice in each group. Data are presented as means ± SEM of 10 pregnant mice per group. ^*^P *<* 0.05.

Consistent with the findings in PE patients in this study (the α7nAChR positive dMφ immunoreactivity was lower than that in the P group), the percentage of CHAT^+^ dMφ was also inhibited in LPS-induced PE-like mice. LPS-induced inhibition of CHAT expression on dMφ could partially restored by nicotine (**Figure 7**). The effects of nicotine could be largely abolished by α-BGT. As CHAT was synthetase for α7nAChR agonist (Ach) (31), the suppressed expression of CHAT and α7nAChR on dMφ in the LPS-treated mice were both enhanced after nicotine treatment, which indicated activation of α7nAChR-mediated cholinergic anti-inflammatory pathway on dMφ in the LPS and nicotine-treated group.

### Nicotine Suppressed Pro-Inflammatory Macrophage M1 Polarization and Enhanced Macrophage M2 Polarization in Decidual Tissues From LPS-Induced PE-Like Mice

**Figure 8:**
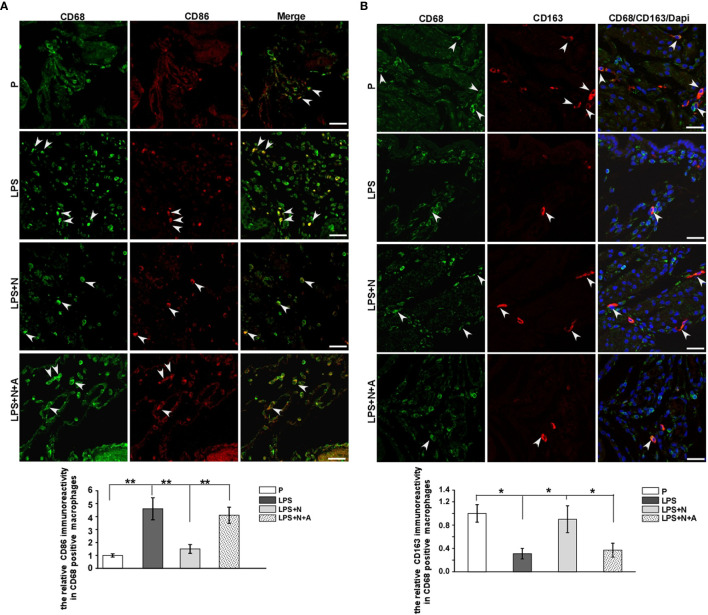
Immunofluorescent staining results showed that nicotine differentially affected the expression of decidual macrophage M1 and M2 biomarkers in PE-like mice. **(A)** Immunostaining of CD86 and CD68 in the decidua from all animal groups and bar graph showed quantitative analysis results on CD86-positive immunoreactivity in dMφ. White arrow heads showed colocalization of dMφ with CD86. Scale bar=30 μm. ^**^P *<* 0.01. **(B)** Immunostaining of CD163 and CD68 in the decidua from all animal groups and bar graph showed quantitative analysis results on CD163-positive immunoreactivity in dMφ. White arrow heads showed colocalization of dMφ with CD163. Scale bar=30 μm. ^*^P *<* 0.05 and ^**^P *<* 0.01.

**Figure 9:**
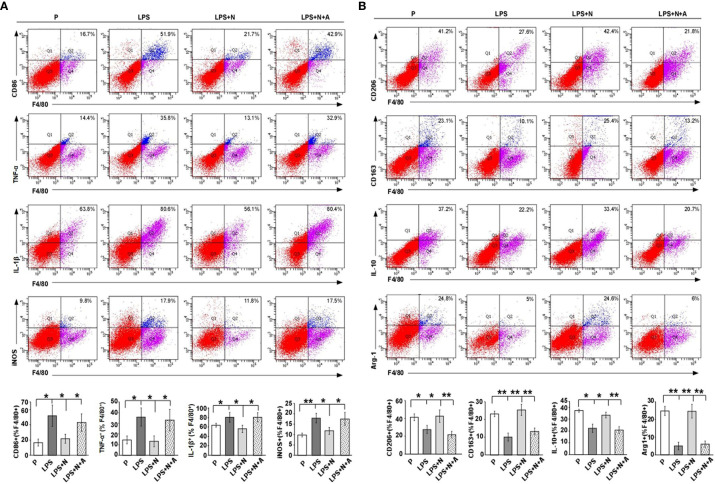
FCM analysis results showed that nicotine prompted the polarization of M1 dMφ to M2 dMφ from LPS-induced PE-like mice. **(A)** FCM analysis of the percentage of CD86 ^+^, TNF-α^+^, IL-1β^+^ and iNOS^+^ dMφ from different animal groups (n=10 each). Total leukocytes were gated using FSC vs. SSC and then gated for identifying CD68^+^ macrophages. Gating strategy was the same to that used to identify CD68^+^ CHAT^+^ cells. **(B)** FCM analysis of the percentage of CD206 ^+^, CD 163^+^, IL-10^+^ and Arg-1^+^ dMφ from different animal groups (n=10 each). ^*^P *<* 0.05 and ^**^P *<* 0.01.

To determine the *in vivo* effects of nicotine on macrophage polarization, we examined the expression of the typical biomarkers for macrophage polarization by immunostaining (**Figure 8**) and FCM (**Figure 9**). CD86, TNF-α, IL-1β and iNOS served as M1 markers, CD163, CD206, IL-10 and Arg-1 served as M2 markers. LPS increased the immunoreactivity of CD86 (M1 marker) and decreased that of CD163 (M2 marker) on dMφ in pregnant mice, while nicotine selectively reduced the expression of CD86 and enhanced the expression of CD163 on dMφ (**Figure 8**). To further examine the effects of nicotine on macrophage M1/M2 polarization in decidual tissues, we determined the expression levels of the typical macrophage biomarkers by FCM. As shown in **Figure 9**, LPS significantly elevated the percentage of CD86 ^+^, TNF-α^+^, IL-1β^+^ and iNOS^+^ dMφ (M1 subtype) but diminished the percentage of CD206 ^+^, CD 163^+^, IL-10^+^ and Arg-1^+^ dMφ (M2 subtype) in pregnant mice. Nicotine treatment decreased the elevation in expression of CD86, TNF-α, IL-1β and iNOS on dMφ induced by LPS, and increased the expression of CD206, CD 163, IL-10 and Arg-1 on dMφ. α-BGT could largely blocked the effects of nicotine. Our study suggests that α7nAChR can regulating the excessive inflammation at the maternal-fetal interface possibly by polarizeM1 dMφ toward M2 dMφ.

## Discussion

Epidemiological studies have shown that smoking can reduce the risk of preeclampsia (32, 33). Cigarette extract (mainly nicotine, often used as α7nAChR agonist) could promote the proliferation and migration of HTR-8/svneo trophoblast (34). Nicotine also alleviated maternal hypertension and proteinuria in LPS-induced PE-like model (18), here we further observed placental pathology including disorder of trophoblast arrangement, narrowed and deformed vessel lumens and more vascular smooth muscle in the LPS-treated group. Nicotine restored the suppressed angiogenic activity of endothelial cells induced by soluble fms-like tyrosine kinase 1(sFlt1) which was closely related to preclamptic outcomes (35). Based on our results and others, pharmacologic intervention of nicotine or its analogues provide new strategies in prevention and treatment of PE.

α7nAChR dysfunction contributes to hypertension disorders associated with inflammation. More severe heart and kidney damage, and higher serum levels of proinflammatory cytokines were observed in α7nAChR^−/−^ mice rendered hypertensive through 2-kidney-1-clip surgery than in wild-type mice (36). The decreased α7nAChR mRNA expression in peripheral blood monocytes (PBM) from PE women was associated with exaggerated inflammation (37). Yoshikawa et al. found that pretreatment with nicotine suppressed the production of the proinflammatory mediators such as TNF-α, COX-2 and macrophage inflammatory protein (MIP)-1α in LPS-activated human PBM (38). α7nAChR activation by nicotine reduced the release of TNF-α in LPS-treated microglial cells (39). In addition, results from Teng et al. found that nicotine inhibited the MMP-9 expression in murine primary bone marrow derived macrophages; the effects of nicotine were abolished by the selective α7nAChR blocker, methyllycaconitine (40). In the present study, we found that the α7nAChR activity on dMφ was reduced both in PE patients and PE-like mice, which was accompanied by elevated inflammation at the decidua; nicotine treatment attenuated decidual inflammation, which was reversed the specific α7nAChR antagonist α-BGT. All the above findings supports the existence of a decidual cholinergic anti-inflammatory pathway mediated by α7nAChR that could be potentially exploited for novel treatments of PE in which local inflammation in decidua, sustained by over activated macrophages, plays a crucial role.

α7nAChR is the major receptor on macrophages in the cholinergic anti-inflammatory pathway, its anti-inflammatory mechanisms are varied under different pathological conditions. α7nAChR stimulation by nicotine could induce prostaglandin E2 (PGE2) expression to increase the protein kinase A (PKA) activity to inhibit IL-12 and TNF-α production in human peripheral blood mononuclear cells (41). Other downstream signaling pathways or proteins from α7nAChR such as JAK2/STAT3 (42) and PI3K/Akt (40) and interleukin-1 receptor-associated kinase M (IRAK-M) played an important role in the anti-inflammatory effect of nicotine on LPS-treated macrophages (43). Up-regulation of TIPE2 (a new member of tumor necrosis factor-α-induced protein-8 family) induced by nicotine through α7nAChR activation also contributed to cholinergic anti-inflammatory effect (44). These studies provide cues in investigating the mechanisms of α7nAChR regulating decidual macrophage polarization in PE, more studies will be needed to clarify the downstream signaling molecules from α7nAChR in PE in the future.

A decreased frequency of dMφ with an M2 phenotype and an increase in the M1 phenotype existed in miscarriage patients (11). In pregnancy-induced hypertension (PIH), higher mRNA levels of TNF-α and IL-1β, lower mRNA levels of IL-4, IL-10, and IL-13 were observed in macrophages differentiated from isolated peripheral blood mononuclear cells than that in normotensive pregnancies (45); the percentage of CD86-positive macrophages (M1) was higher and percentage of CD163-positive macrophages (M2) was lower in PIH group than that in control group (45). In PE patients, by using immunohistochemistry method, Schonkeren et al. found a significant lower number of M2 dMφ (CD163^+^CD14^+^ ratio) from preterm preeclamptic pregnancies compared with preterm control pregnancies (10); consistent with this study, we found that both the PE patients and PE-like animals induced by LPS presented a significant higher proportion of M1 dMφ and a lower proportion of M2 dMφ by using double immunofluorescence method and FCM analysis. Furthermore, nicotine treatment inhibited a shift to the M1 dMφ in the LPS-induced PE model and lowered the excessive inflammation at the maternal-fetal interface. Such effects of nicotine could be largely abolished by α7nAChR antagonist (α-BGT). The potential protective role of nicotine in PE is related to α7nAChR mediating the polarization of M1 macrophages to M2 macrophages in decidua. Indeed, LPS could induce BV-2 microglia (a type of macrophage) into M1 subtype, while α7nAChR agonist (acetylcholine, Ach) could suppress the release of pro-inflammatory factors and promote the release of anti-inflammatory factors; α7nAChR knockdown block the effects of Ach (46). However, further investigations are needed to reveal the detailed mechanisms underlying α7nAChR-medited the polarization of M2 dMφ in PE.

Several studies proposed other signaling pathways were involved in the imbalance of dMφ polarization. T cell immunoglobulin mucin 3 (Tim-3) interacts with its ligand Galectin-9 (Gal-9) to induce immune tolerance (47). PE-like impairment in pregnant rats was alleviated by Tim-3/Gal-9 through prompting dMφ polarization to M2 phenotypes (48). Macrophage colony-stimulating factor (M-CSF) is a differentiating factor, M-CSF secreted by cultured leukocyte-free first-trimester decidual cells could induce M2 macrophage polarization after proinflammatory stimuli (49). Results observed in samples from PE patients in combination with that found in samples from PE-like model in this study showed that stimulation of α7nAChR by nicotine suppressed decidual M1 macrophage polarization against excessive inflammation in PE. However, further *in vitro* research is needed to elucidate such findings.

In conclusion, abnormal low α7nAChR activity on dMφ along with a decrease in M2 dMφ and an increase in M1 dMφ existed in human PE and the mouse model of PE induced by LPS, which indicated that the suppression of α7nAChR might result in preeclampsia possibly through the M1 dMφ-triggered excessive maternal–fetal inflammatory response. In addition, stimulation of α7nAChR on dMφ by nicotine attenuated LPS-induced pathological structures of placenta and decidual inflammation. This protective effect of nicotine can be associated with the enhancement of the polarization of M1 dMφ to M2 dMφ. This study provides novel evidence supporting the future development of therapeutic target for PE.

## Data Availability Statement

The original contributions presented in the study are included in the article/supplementary material. Further inquiries can be directed to the corresponding author.

## Ethics Statement

The studies involving human participants were reviewed and approved by The Ethics Committee of the Guangzhou women and children’s medical center (No. 2018041701 and No. 201922200). The patients/participants provided their written informed consent to participate in this study. The animal study was reviewed and approved by the Guangzhou Medical University Animal Ethics Committee (Permit Number: 2012–50).

## Author Contributions

ZZ, BZ, and BL collected samples. XH, WL, and PL conducted the experiments. XH, WL, PL, KG, PH, and JY analyzed the data. XH and JY conceived and designed the experiments as well as wrote the paper. WL participated in the key experiment supplement of immunofluorescence double labeling and statistical data analysis. PH participated in the revision of the whole article, and participated in the polishing of the article, and provided some financial support in the process of revision. All authors contributed to the article and approved the submitted version.

## Funding 

This study was supported by the National Natural Science Foundation of China (81971417, 81901568 and 81801466).

## Conflict of Interest

The authors declare that the research was conducted in the absence of any commercial or financial relationships that could be construed as a potential conflict of interest.

The reviewer WW declared a shared affiliation with the authors to the handling editor at the time of review.
